# Directional terahertz holography with thermally active Janus metasurface

**DOI:** 10.1038/s41377-023-01177-4

**Published:** 2023-06-05

**Authors:** Benwen Chen, Shengxin Yang, Jian Chen, Jingbo Wu, Ke Chen, Weili Li, Yihui Tan, Zhaosong Wang, Hongsong Qiu, Kebin Fan, Caihong Zhang, Huabing Wang, Yijun Feng, Yunbin He, Biaobing Jin, Xinglong Wu, Jian Chen, Peiheng Wu

**Affiliations:** 1 Research Institute of Superconductor Electronics (RISE), School of Electronic Science and Engineering, Nanjing University, Nanjing, 210023 China; 2 Purple Mountain Laboratories, Nanjing, 211111 China; 3 Ministry-of-Education Key Laboratory for the Green Preparation and Application of Functional Materials, Hubei Key Laboratory of Polymer Materials, School of Materials Science and Engineering, Hubei University, Wuhan, 430062 China; 4 School of Electronic Science and Engineering, Nanjing University, Nanjing, 210023 China; 5 National Laboratory of Solid State Microstructures and School of Physics, Nanjing University, Nanjing, 210093 China

**Keywords:** Terahertz optics, Metamaterials

## Abstract

Dynamic manipulation of electromagnetic (EM) waves with multiple degrees of freedom plays an essential role in enhancing information processing. Currently, an enormous challenge is to realize directional terahertz (THz) holography. Recently, it was demonstrated that Janus metasurfaces could produce distinct responses to EM waves from two opposite incident directions, making multiplexed dynamic manipulation of THz waves possible. Herein, we show that thermally activated THz Janus metasurfaces integrating with phase change materials on the meta-atoms can produce asymmetric transmission with the designed phase delays. Such reconfigurable Janus metasurfaces can achieve asymmetric focusing of THz wave and directional THz holography with free-space image projections, and particularly the information can be manipulated via temperature and incident THz wave direction. This work not only offers a common strategy for realizing the reconfigurability of Janus metasurfaces, but also shows possible applications in THz optical information encryption, data storage, and smart windows.

## Abstract (web summary)

A thermally active terahertz Janus metasurface with direction dependence and reconfigurability was reported. Bidirectional and dynamic control of electromagnetic functions, such as asymmetric meta-lens and directional holographic imager, were achieved.

## Graphical abstract

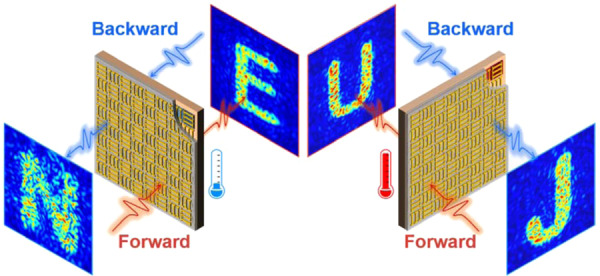

## Introduction

In the past decades, metasurfaces have drawn tremendous attention due to their unprecedented abilities to manipulate electromagnetic (EM) waves at will^1–3^. With the development of compact and integrated systems, there are increasing demands on integrating multiple functionalities into a single metasurface. The wave propagation direction is a vital wave property and its multiplexing endows the metasurface with rich functions in two asymmetric channels. The requirement of asymmetric wave manipulation widely exists in practical applications such as laser technologies^4,5^, antenna radomes^6,7^, full-duplex communication^8,9^, *etc*. However, most existing multifunctional metasurfaces do not exploit this design freedom. Although nonreciprocal devices could be created to realize direction-dependent manipulation of EM waves by breaking the time-reversal symmetries using magneto-optic materials^10,11^, nonlinear materials^12,13^, and time-variant materials^14,15^, the apparatus used for external control is always complicated and bulky.

Recently, asymmetric but still reciprocal transmission in opposite propagation directions using symmetry-broken structures has been reported^16^, showing unconventional wavefront tailoring in a direction-determined manner. The Janus metasurface consisting of a stack of twisted meta-sheets can act as free-space holographic imagers for opposite propagation directions. Compared to conventional metasurface holography, the additional freedom brought by the propagation direction in Janus metasurface can further enhance the design space and information capacity. Towards practical applications of direction-dependent metasurfaces, the reconfigurability with the change of external incentives is highly expected^17–21^. However, due to the difficulty in designing and fabricating the devices integrated with active components, EM functionalities of the Janus metasurface are always static, limiting their application prospects^22–28^.

The manifold spectrum resources and the compactness of the system make THz wave promising in applications such as high-capacity data communication and high-security EM encryption^29–31^. Multifunctional THz devices capable of dynamically reconfiguring the function are essential for further enhancing the data storage capacity and expanding application scenarios. Recently, based on various tunable materials, reconfigurable and programmable metasurfaces have achieved the multiplexing of incident polarization, frequency and angle^18,32–35^. However, how to realize direction-dependent and reconfigurable THz metasurface is still an untapped area.

Here, we studied the directional THz holography using a proposed reconfigurable Janus metasurface. The proposed metasurface based on vanadium dioxide (VO_2_) shows thermally controlled reconfigurability and incident direction dependence, thus manifesting four independent EM functionalities in the whole space. Therefore, in applications of zoom lenses, such a reconfigurable Janus metasurface can generate four different focal points actively controlled by propagation directions or temperature. Moreover, the reconfigurable direction-dependent meta-hologram capable of generating four different images is experimentally demonstrated. The proposed active direction-multiplexing strategy provides a new solution in optical data storage and encryption applications where the multiplexing technique is urgently required to improve system capacity and integration.

## Results

### Concept design

**Fig. 1:**
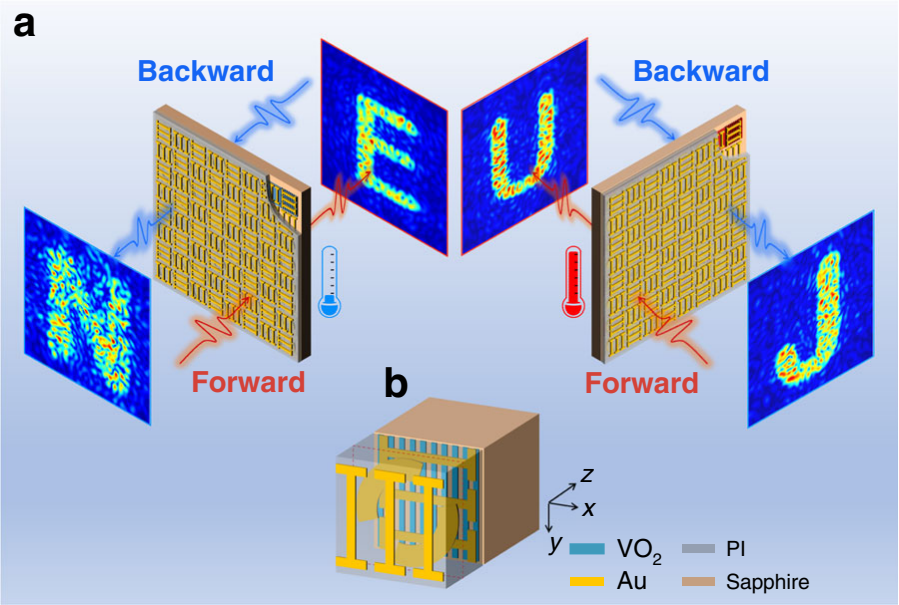
Schematics of the reconfigurable THz Janus metasurface. **a** Four holographic images, “N”, “J”, “U”, and “E”, independent of each other, were reconstructed from four independent phase profiles which are all encrypted on one metasurface with keys of incident direction and VO_2_ state. **b** Schematic of a VO_2_-integrated active meta-atom with cascaded metallic structures

Figure 1a illustrates the concept of reconfigurable Janus metasurface. The designed meta-atoms have asymmetric transmission characteristics for linearly polarized (LP) waves (e.g., *x*-polarization). The two spatial phase profiles for the same-polarized incident wave from two opposite directions were calculated based on Gerchberg–Saxton (GS) algorithm. They are realized by combining two kinds of unidirectional meta-atoms, one for forward and the other for backward direction, that are rotated to each other by 90 degrees in the *x*–*y* plane. The meta-atoms are arranged into chessboard-like distributions and selectively perform the desired wave functionalities depending on the incident direction. Hence, the metasurface shows two independent spatial phase profiles for the same polarized THz wave from the forward and backward incidence. Moreover, due to the reversible insulator-metal-transition (IMT) of VO_2_, the metasurface can dynamically switch its spatial phase profiles for each incident direction. Thereby, two different holographic images can be generated before and after the IMT of VO_2_ for each incident direction.

The critical step to implementing active Janus metasurface is the construction of meta-atoms with both unidirectional transmission and phase modulation. Here, we employed the meta-atom with cascaded anisotropic metallic structures, as shown in Fig. 1b. The three metallic layers are separated by 10 μm-thick polyimide film and stacked along the propagation direction. The top and bottom metallic layers are wire grating structures perpendicular to each other. The metallic structures in the middle layer are crucial to achieving high-efficiency linear polarization conversion with specific phase delays. A group of metallic structures with a full 2π phase coverage and asymmetric transmission was obtained through a proper choice of geometric structure and parameter optimization (see more details in Supplementary Section 1). Utilizing the IMT of VO_2_ film, we achieve thermally controlled reconfigurability. The VO_2_ wire grid structure made from 200 nm-thick VO_2_ film is patterned on the sapphire substrate, which is connected vertically to the metallic grating on the bottom layer.

**Fig. 2:**
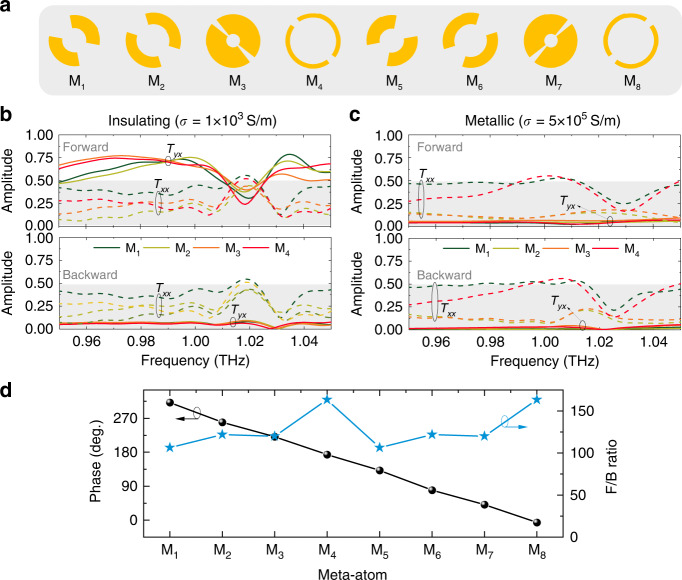
Simulated transmission spectra of meta-atoms in THz Janus metasurface. **a** Schematic diagrams of the eight SSRRs in meta-atoms of M_1_-M_8_. **b**, **c** Simulated cross/co-polarized transmission spectra of M_1_-M_4_ under the normal incidence of *x*-polarized THz wave. The VO_2_ conductivities in the insulating and metallic states are set to be 1 × 10^3 ^S/m (**b**) and 5 × 10^5 ^S/m (**c**), respectively. The meta-atoms of M_5_-M_8_ have the same transmission amplitude as M_1_-M_4_. Hence, only the transmission spectra of M_1_-M_4_ are shown here. **d** Cross-polarized transmission phase of the eight meta-atoms under the forward incidence of *x*-polarized THz wave at 0.99 THz when VO_2_ is in the insulating state. Phase differences between adjacent meta-atoms are about π/4. The F/B ratio is defined as the ratio of the cross-transmission at forward incidence to that at backward incidence

In our design, we constructed eight different dual-gap symmetric split-ring resonators (SSRRs) in the middle layer (the corresponding meta-atoms are named M_1_-M_8_), and the phase spacing between each meta-atom is around π/4. For simplicity, after rotating the SSRR of M_1_-M_4_ by 90° in the *x*–*y* plane, M_5_-M_8_ are obtained correspondingly, and an additional phase delay of π is added to the cross-polarized transmission phase. The optimized SSRRs are shown in Fig. 2a, and the geometric parameters are listed in Table S1 in Supplementary Materials.

The simulated normalized cross/co-polarized transmittance spectra of the VO_2_-integrated meta-atoms are plotted in Fig. 2b, c. On the whole, we discuss transmission properties of the *x*-polarized incident waves for both forward and backward incidences. The results for *y*-polarized incidence can be derived according to the reciprocity theorem (see details in Supplementary Material). The cross-transmission amplitudes range from 0.65 to 0.75 for the eight meta-atoms when the VO_2_ is in the insulating state (Fig. 2b). After the IMT of VO_2_ caused by the temperature rise from 25°C to 85°C, VO_2_ wire grids go into the metallic state. Then, the metallic grating on the bottom layer is shortened by VO_2_ grids, resulting in a significant reduction of the cross-polarized transmission coefficient, as plotted in Fig. 2c. For the forward incidence (along the *z*+ direction), the cross-polarized transmission phase of the designed meta-atoms around 0.99 THz fully cover the 2π phase range with a step of ~π/4, as shown in Fig. 2d. For the backward incidence, the cross-polarized transmission coefficients are closed to zero no matter whether VO_2_ is in the insulating or metallic state, as seen in the bottom figures of Fig. 2b, c.

In the designed meta-atoms, part of the transmitted wave is converted to its orthogonal polarizations with respect to the incident wave. The huge linear polarization conversion efficiency difference for the opposite propagation directions leads to asymmetric transmission. Here, the forward-to-backward ratio is introduced to evaluate the performance of the asymmetric transmission. It is defined as |${T}_{yx}^{f}$|^2^/|${T}_{yx}^{b}$|^2^, where ${T}_{yx}^{f}$ is the cross-transmission coefficient illuminated by the *x*-polarized wave from the forward direction while ${T}_{yx}^{b}$ is for the backward incidence case (along the *z*− direction). As shown in Fig. 2d, |${T}_{yx}^{f}$|^2^/|${T}_{yx}^{b}$|^2^ for eight meta-atoms are always >100 at the target frequency when VO_2_ is in the insulating state, indicating a remarkable transmission difference along the opposite directions.

### Dynamic Janus meta-lens

Using the meta-atoms featuring asymmetric transmission and phase manipulation, we construct dynamic Janus metasurfaces to demonstrate their versatile optical functionalities, including zoom lenses with reconfigurable focal points and direction-dependent holography at THz frequencies. In the first demonstration, we develop a metasurface with a reconfigurable focus for the two incident directions. The focal point for the forward wave moves in a cross-section perpendicular to the propagation direction. For example, before the IMT of VO_2_, the focal point is located on the position of *L*_1_ (*x*, *y*, *z*) = (−1.50 mm, 0, 3.00 mm) and is switched to the position of *L*_2_ (+1.50 mm, 0, 3.00 mm) after the IMT of VO_2_. Meanwhile, the focal distance for the backward wave can be tuned by VO_2_ conductivity.

**Fig. 3:**
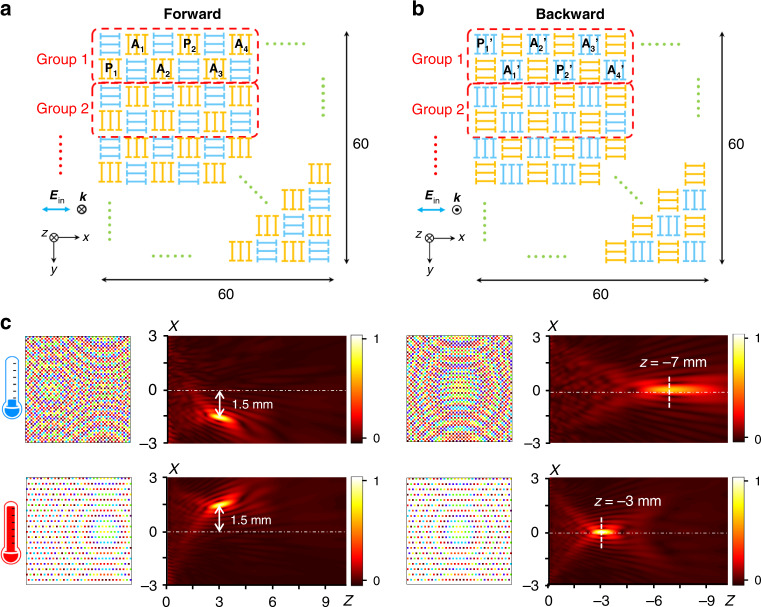
THz Janus meta-lens for the reconfigurable and direction-dependent focus of THz wave. **a** Schematic of the top-layered metallic pattern. In the forward incidence of the *x*-polarized wave, only the meta-atoms with a top-layered pattern perpendicular to the polarization direction modulate the cross-transmission. Those meta-atoms are colored yellow, while others are colored blue. The metasurface consists of 60 × 60 meta-atoms. The periodicity of the meta-atom is 100 μm. **b** Schematic of the bottom-layered metallic pattern. The meta-atoms working for backward incidence in each group are denoted as P_1_', P_2_', A_1_', A_2_', A_3_', and A_4_'. **c** Spatial phase profiles of the metasurface and numerically simulated electric field distributions for the *y*-LP transmitted THz wave, which is obtained based on the Rayleigh-Sommerfeld diffraction integral formula

The design principle of reconfigurable Janus meta-lens is schematically shown in Fig. 3a, b. The metasurface comprises 60 × 60 meta-atoms, giving an overall size of 6 mm × 6 mm. Half of the meta-atoms generate the phase profiles for the forward (yellow) and backward (blue) waves, respectively, as shown in Fig. 3a, b. The 60 × 60 meta-atoms are divided into 300 groups, each consisting of 2 × 6 meta-atoms. The six meta-atoms for the same incidence are labeled with ‘P_1_’, ‘P_2_’, ‘A_1_’, ‘A_2_’, ‘A_3_’, and ‘A_4_’, respectively. P_1_ and P_2_ are passive meta-atoms, as they are without VO_2_ active elements. The other meta-atoms integrated with VO_2_ elements are active meta-atoms. The passive meta-atoms have a slightly higher transmission efficiency over active ones because the resistive VO_2_ film is removed (see more details in Supplementary Section 2). When VO_2_ is in the metallic state, the cross-polarized transmittance of the active meta-atoms is reduced to nearly zero, leaving only P_1_ and P_2_ to manipulate the wavefront of the *x*-polarized forward wave.

Then, based on the geometric optical principle^36^, to focus the incident wave at a focal point of *L*_2_ (+1.50 mm, 0, 3.00 mm), the phase shifts provided by meta-atoms of P_1_ and P_2_ should satisfy:

1 $$\varphi \left(x,\,y\right)=\frac{2\pi }{\lambda }\left[\sqrt{{\left(x-{x}_{{L}_{2}}\right)}^{2}+{\left(y-{y}_{{L}_{2}}\right)}^{2}+{z}_{{L}_{2}}^{2}}-{z}_{{L}_{2}}\right]$$

where *λ* is the wavelength of the incident THz wave in the free space, (*x*, *y*) is the position of P_1_ and P_2_ at the plane of *z* = 0, (${x}_{{L}_{2}},\,{y}_{{L}_{2}},\,{z}_{{L}_{2}}$) is the position of the focal point of *L*_2_. When VO_2_ returns to the insulating state, the function of A_2_ and A_4_ is to erase the focal point of *L*_2_. Their complex cross-polarized transmission coefficients are designed to have the same amplitude but a phase shift of π relative to P_1_ and P_2_. Meanwhile, a new focal point of *L*_1_ can be formed arbitrarily using Eq. 1, which is determined by the secondary field radiations with specific phase profiles generated by A_1_ and A_3_ under the illumination of the same polarized wave. Following the above strategy, the active Janus meta-lens can switch the focal point transversely for the forward wave. A similar approach is adopted to achieve an adjustable focal length for the backward wave (see detailed description in Supplementary Section 3).

Figure 3c illustrates phase profiles of the metasurface in two opposite incident directions before (top panels) and after (bottom panels) the IMT of VO_2_. In the phase profiles of Fig. 3c, the meta-atoms with zero cross-transmission are colored white. Based on the Rayleigh-Sommerfeld diffraction integral formula^37^ (see details in Methods), we numerically calculated the THz diffracted field distributions on the *xOz* plane. In the calculation, the transmission amplitude of the meta-atoms contributing to the focal point is set to 1. The results at 0.99 THz are shown in Fig. 3c. For the forward incidence, the focal point moves along the *x*-direction with the phase change of VO_2_, verifying that two independent phase profiles are well multiplexed on a metasurface. For the backward incidence, another two profile phases are combined into the metasurface, which determines the longitudinal shift of the focal length.

**Fig. 4:**
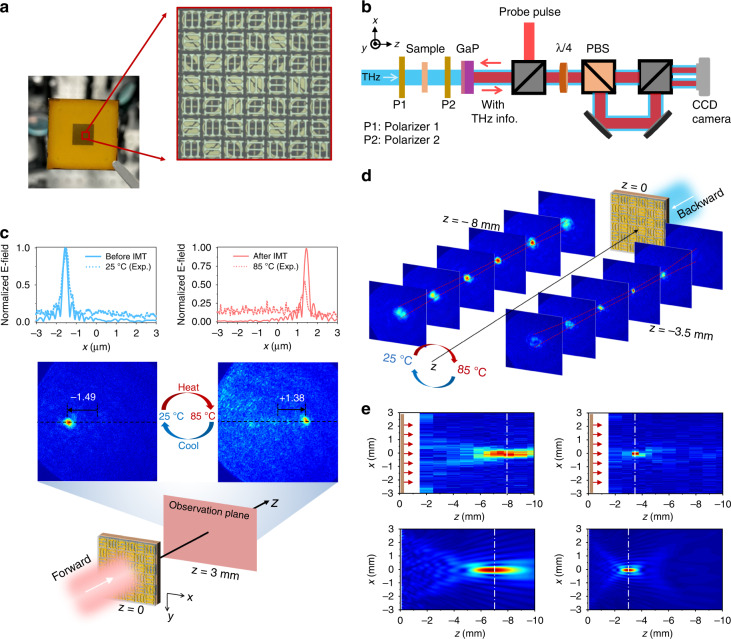
Experimental demonstration of the reconfigurable Janus metasurface as THz meta-lens. **a** Microscopic image of the fabricated reconfigurable Janus metasurface. **b** Diagram of the imaging part of the THz spectral imaging system. **c** Imaging results obtained at the observation plane of *z* = 3.0 mm at 1.01 THz for the forward waves. At 25 °C, the *y*-LP transmitted wave is focused at (−1.49 mm, 0, 3 mm), and the focal point moves to (+1.38 mm, 0, 3 mm) when temperature increases to 85 °C. The insets show the *x*-cut normalized electric field profile at *z* = 3.0 mm, the dashed lines are the measured electric field along with the middle horizontal line in the graph below it, and the solid lines are numerically calculated using Rayleigh-Sommerfeld integral formula. **d** Measured imaging results at different observation planes for the backward waves. The focal length of the fabricated metasurface is varied from −8.0 mm to −3.5 mm by controlling VO_2_ conductivity. **e** Normalized electric field profile at the *x*O*z* plane. The measured results (top) are extracted from the electric field slices in (**d**), and the numerical results (bottom) are calculated by using the integral formula

In the following, we fabricated the designed metasurface (see Methods for fabrication process), and Fig. 4a shows the microscopic image of the fabricated sample. We characterized the sample using the THz spectral imaging system (Fig. 4b) under *x*-polarized incidence (see Methods for details). Figure 4c–e illustrates the measured *y*-polarized field distributions of the sample at 1.01 THz at different temperatures. When the sample is at room temperature of 25 °C, and the sample is illuminated from the forward incident wave, the synergy of the active and passive meta-atoms results in the generation of a hyperboloidal phase profile to focus the output *y*-polarized wave at the designed position.

As shown in Fig. 4c, the *y*-polarized field distributions at the plane of *z* = 3.00 mm prove that the focal spot is at (−1.49 mm, 0, 3.00 mm). We draw the *x*-directional cut of the measured focal spot profiles to compare the experimental and numerical results. The experimental and numerical values are normalized with their maximum values. In our experiment, the metasurface can focus the forward wave at (−1.49 mm, 0 mm, 3.00 mm) at 25 °C and the focal point moves to (+1.38 mm, 0 mm, 3.00 mm) when the temperature increases to 85 °C, showing good agreement with the numerical calculation. However, the normalized E-field amplitude experimentally obtained at 85 °C is distinctly smaller than the calculated values, mainly because A_2_ (A_4_) is not completely switched off when VO_2_ goes into the metallic state. In that case, only a portion of the THz wave leaks out and coherently cancels out the diffracted fields generated by P_1_ (P_2_) at the focal point of *L*_1_ (see detailed theoretical analysis in Supplementary Section 5).

We measured the transmitted (*y*-LP) electric field distributions for the backward waves by moving the sample along the *z*-direction. As shown in Fig. 4d, the red dash lines depict the trajectories of the full-width at half-maximum (FWHM) as a function of distance along the *z*-direction. As the sample is heated from 25 °C to 85 °C, the focal length of the sample changes from −8.0 mm to −3.5 mm, while the corresponding theoretical values are −7.0 mm and −3.0 mm, respectively (Fig. 4e). Figure 4e depicts the measured and calculated normalized (*y*-LP) field distributions on the *xOz* plane. Since the depth of focus is on the order of millimeters, the slight deviation between the experiments and numerical results is within the uncertainty of measurement.

### Dynamic Janus meta-holograms

In the following, we designed a reconfigurable Janus metasurface capable of producing direction-dependent and temperature-dependent holographic images. Similar to the first demonstration, the meta-atoms for the forward and backward incidences are arranged in a chessboard pattern. The phase distributions of these meta-atoms required for the holographic images are calculated based on the GS algorithm. To realize the switching of holographic images, we divide the meta-atoms into groups and arrange the active and passive meta-atoms in a specific rule similar to the meta-lens (see details in Supplementary Section 3).

**Fig. 5:**
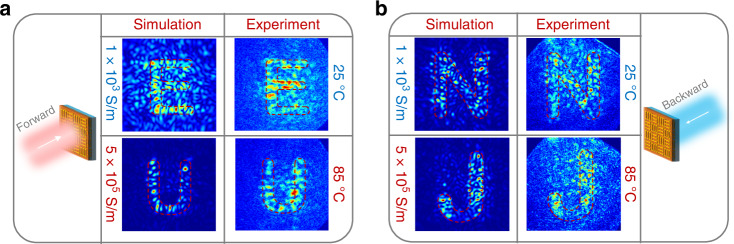
Holographic images generated from the reconfigurable Janus metasurface. **a**, **b** Simulated and measured field distributions at 25 °C and 85 °C, respectively, for the forward (**a**) and backward (**b**) incident waves. Simulated field distributions are obtained at VO_2_ conductivities of 1 × 10^3^ and 5 × 10^5 ^S/m, respectively

As an illustrative example of the active Janus meta-hologram, the imaging targets are set to four letters in the four independent projecting channels. We performed a full-wave simulation to achieve the diffracted fields of the designed metasurface. The VO_2_ conductivity before and after the IMT are 1 × 10^3 ^S/m and 5 × 10^5 ^S/m, respectively. The simulated *y*-LP field distribution at the plane of *z* = ±2.10 mm at *f* = 1.00 THz is plotted in Fig. 5. To verify its functionality, we fabricated the metasurface consisting of 60 × 60 meta-atoms with a total size of 6 mm × 6 mm.

Figure 5 illustrates the measured *y*-LP transmission field distribution at the observation plane when the 1.00 THz wave is incident from the two opposite directions. The slight difference in the working frequency between meta-lens and meta-hologram is mainly attributed to the fabrication error in the thickness of the polyimide layer. The distance between the observation plane and the metasurface is 2.20 mm for both the forward and backward incidences. In Fig. 5a, when the metasurface is illuminated from the front, the holographic image can switch from “E” to “U” as the metasurface is heated from 25 °C to 85 °C. When the metasurface is illuminated from the back, the generated holographic images can be reversibly switched between “N” and “J”, as illustrated in Fig. 5b. Therefore, the direction multiplexing and reconfigurability of the meta-hologram are experimentally demonstrated. The simulated images show good accordance with the experimental results. The slight deviation between the experimental and simulated results can be attributed to layer misalignments and imperfections in the fabrication process. There is a slight difference between the simulated and the numerically calculated images (see Supplementary Materials for details), probably because the VO_2_ loss in the active meta-atoms and the coupling interactions between the adjacent meta-atoms distort the amplitude and phase distribution from the designed one.

### Information encryption

**Fig. 6:**
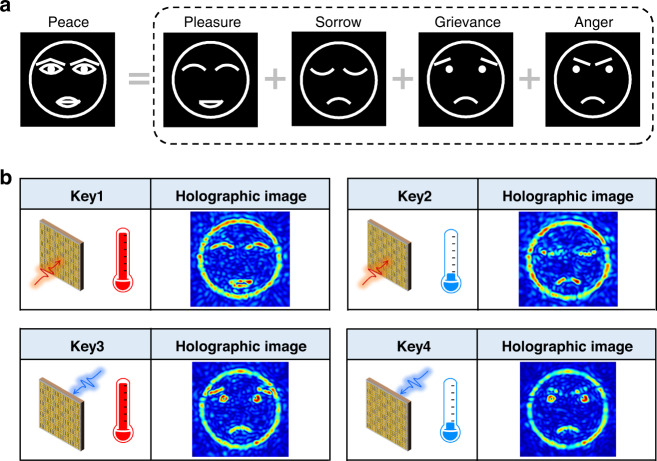
Security-sharing function based on the reconfigurable Janus metasurface. **a** Emoji of “peace” stored in the metasurface as the secret, and it can be decomposed into four other emojis of “pleasure,” “sorrow,” “grievance,” and “anger”. **b** Holographic images of the four emojis decrypted from the metasurface with keys of the incident direction and temperature

Information encryption can be achieved by splitting a secret among multiple shareholders, for example, dividing the target image into several parts, so the eavesdropping of a single channel will not leak true information about the shared secret. The proposed metasurface, capable of generating different and independent holographic images, can be applied to information encryption. Herein, we demonstrated the effectiveness of the security-sharing function based on our proposed metasurface. As shown in Fig. 6a, a “peace” emoji is to be stored in the reconfigurable Janus metasurface as the secret, and the emoji is decomposed into four different emojis, i.e., “pleasure”, “sorrow”, “ grievance,” and “anger”. These emojis are encrypted to different channels of the same metasurface. Figure 6b shows the holographic images of the four emojis decrypted from the proposed metasurface with keys of the incident direction and temperature. In that case, the eavesdropping of a single channel will not leak the true secret of the “peace” emoji. Instead, only the misleading information is wiretapped if the data in the total four channels are not decrypted simultaneously. From this perspective, the proposed metasurface can significantly improve information security.

## Discussion

In our demonstration, the meta-lens show rich varifocal properties. Hence, it offers many potential applications in THz imaging, such as extending the depth of field^38,39^. The multiplexing demonstrated in our meta-hologram is essential to enhancing the capacity of the holographic plate. The holographic targets are not limited to what has been demonstrated herein, and the additional degrees of freedom enabled by the direction and temperature could further enhance information security because the eavesdropping of the information should be done at physical levels with multiple independent channels.

The thermal switching time in our experiment is on the order of minutes. Introducing the electrical or optical switching methods will significantly improve the switching speed^40–42^. Besides, the transmission efficiency of the reconfigurable Janus metasurface can be further optimized through removing the sapphire substrate or replacing it with the flexible substrates with low refractive index^43–45^. Recently, the electrical addressability and non-volatile memory effect of metasurface based on phase change material have been reported^19,41,46–50^. If the above functions are introduced into the Janus metasurface, the meta-lens and meta-holography can be reconfigured more flexibly. In that case, the information capacity and application scenarios will increase dramatically. Moreover, the reconfigurable Janus metasurface may be realized by other tunable materials, such as liquid crystal^34,51,52^, germanium antimony telluride (GST)^53–55^ or hydrogenated/dehydrogenated magnesium^32,56^. A similar reconfigurable direction-dependent metasurface can be developed based on the proposed scheme and fulfill multiple functionalities in other frequency bands.

In summary, we proposed a thermally active Janus metasurface scheme in which the propagation direction of THz waves can be multiplxed, and the functionality for each direction can be reconfigured. For proof-of-concept demonstrations, we designed and fabricated reconfigurable THz Janus meta-devices, including asymmetric meta-lens and directional holographic imager. The experimental results demonstrate their different THz functionalities can be switched by changing the incidence direction and temperature. The metasurface shows numerous potential applications such as optical information encryption, data storage, wireless communication, and holography.

## Materials and methods

### Device fabrication

A 200-nm-thick VO_2_ film was grown on (0001)-sapphire substrate by pulse laser deposition^57^. Then, a positive photoresist film (AZ1500) was spin-coated on the sample, and the wire grid pattern was formed by ultraviolet photolithography. The pattern was transferred to the VO_2_ film via reactive ion etching with a CF_4_ gas flow rate of 40 sccm and a radio frequency power of 100 W. Next, the bottom-layered pattern was imprinted onto the photoresist film (AZ1500) after the second ultraviolet photolithography. The 10-nm-thick titanium and 200-nm-thick gold films were sputtered on the sample by magnetron sputtering, and the bottom-layered gold structures were formed after the lift-off process. Then, the polyimide solution with a viscosity of 3600 cp was spin-coated on the sample, followed by baking the sample at 90 °C, 120 °C, 180 °C, 220 °C and 250 °C for 0.25, 0.5, 0.5, 0.5 and 2.5 h, respectively. The above processes are repeated to form other layers of gold structures and another polyimide layer. A detailed flow chart of the fabrication process is illustrated in Fig. S4.

### THz imaging measurement

The diffracted E-field of the proposed Janus metasurface was measured using a self-built THz spectral imaging system (see details in Supplementary Section 9). THz pulsed radiation was generated in the LiNbO_3_ crystal based on the optical rectification effect, and the collimated wave was incident on the metasurface. The metasurface was fixed on a holder with a 5 mm radius hole to allow the transmission of the THz wave. The resistive heating rods under the holder were used to heat the sample with a temperature controlled by a proportional-integral-derivative (PID) controller. The transmitted THz field distribution was detected by the GaP crystal and a commercial near-infrared charge coupled device (iXon Ultra 888, Oxford Instruments.). A THz polarizer (PW005, PureWavePolarizers Ltd.) was put in front of the metasurface to ensure the incident THz wave was *x*-polarized. A THz polarizer was also put in front of the GaP crystal for detecting the *y*-polarized component of the transmitted wave. The transmitted THz field distribution at different *z*-position was captured by moving the metasurface longitudinally to adjust its distance to the GaP crystal. In the measurement, the entire THz beam path is enclosed in a dry-air purged box (see Fig. S11) to diminish the attenuation of THz waves.

### Numerical analysis of field distribution

The Rayleigh-Sommerfeld diffraction integral formula was used to calculate the field distribution diffracted by the metasurface

2 $$E\left(\xi ,\eta ,{z}_{0}\right)=-\frac{i}{\lambda }\iint {Obj}(x,y,0)\frac{{\rm{exp }}({ik}\left|{\boldsymbol{r}}-{{\boldsymbol{r}}}_{0}\right|)}{\left|{\boldsymbol{r}}-{{\boldsymbol{r}}}_{0}\right|}{\rm{cos }}\delta {\rm{d}}x{\rm{d}}y$$

where *Obj*(*x*, *y*, 0) is the complex transmission coefficient of the reconfigurable metasurface, *k* and *λ* are the wavevector and wavelength in free space, respectively. The item $\left|{\boldsymbol{r}}-{{\boldsymbol{r}}}_{0}\right|$ denotes the distance between (*ξ*, *η*, *z*_0_) and (*x*, *y*, 0). cos(*δ*) is the inclination factor, and it can be denoted by cos(*δ*) ≈ *z*_0_ / |***r*** − ***r***_0_| ≈ 1 under the paraxial approximation. The diagram of Rayleigh-Sommerfeld diffraction on the metasurface is plotted in Fig. S12. Because the proposed metasurface is a phase-only modulator, it can be simplified as follows,

3 $$E\left(\xi ,\eta ,{z}_{0}\right)=-\frac{i}{\lambda }\iint {\rm{exp }}(i\varphi (x,y))\frac{{\rm{exp }}({ik}\left|{\boldsymbol{r}}-{{\boldsymbol{r}}}_{0}\right|)}{\left|{\boldsymbol{r}}-{{\boldsymbol{r}}}_{0}\right|}{\rm{d}}x{\rm{d}}y$$

where $\varphi (x,y)$ is the phase distribution of the reconfigurable Janus metasurface.

## Supplementary information

The SupplementaryInfo for production
