# Supplementary material for: Directional terahertz holography with thermally active Janus metasurface
Source: Light Sci Appl. 2023 Jun 5;12:136. doi: 10.1038/s41377-023-01177-4 (PMC10239755; doi:10.1038/s41377-023-01177-4)
Supplement: Supplementary file 1 — The SupplementaryInfo for production [file 41377_2023_1177_MOESM1_ESM.docx]

# Supplementary Information for

Directional Terahertz Holography with Thermally Active Janus Metasurface

Benwen Chen^1,2^, Shengxin Yang^1^, Jian Chen^3^, Jingbo Wu^1,2*^, Ke Chen^4*^, Weili Li^1,2^, Yihui Tan^1^, Zhaosong Wang^1^, Hongsong Qiu^1^, Kebin Fan^1,2^, Caihong Zhang^1,2^, Huabing Wang^1,2^, Yijun Feng^4^, Yunbin He^3*^, Biaobing Jin^1,2*^, Xinglong Wu^5^, Jian Chen^1,2^, and Peiheng Wu^1,2^

*^1^Research Institute of Superconductor Electronics (RISE), School of Electronic Science and Engineering, Nanjing University, Nanjing 210023, China*

*^2^Purple Mountain Laboratories, Nanjing 211111, China*

*^3^Ministry-of-Education Key Laboratory for the Green Preparation and Application of Functional Materials, Hubei Key Laboratory of Polymer Materials, School of Materials Science and Engineering, Hubei University, Wuhan 430062, China*

*^4^School of Electronic Science and Engineering, Nanjing University, Nanjing 210023, China*

*^5^National Laboratory of Solid State Microstructures and School of Physics, Nanjing University, Nanjing 210093, China*

*Correspondence should be addressed to Jingbo Wu (jbwu@nju.edu.cn), Ke Chen (ke.chen@nju.edu.cn), Yunbin He (ybhe@hubu.edu.cn), and Biaobing Jin (bbjin@nju.edu.cn)*

Section 1. Design and parameter optimization of the meta-atoms.

In the design of the meta-atoms to achieve asymmetric transmission, strong chiral responses for linearly-polarized THz waves are required. Three layers of metallic pattern with a twisted angle between adjacent layers are commonly utilized to break the out-of-plane symmetry, mimicking the construction of helical molecules in nature.

In our design, the gold structures at the top and bottom layers are identical, one of which is rotated by 90° in the *xy* plane. The metallic structures in the middle layer are the dual-gap symmetric split-ring resonators (SSRR), and the orientation of its gap is 45° relative to the *x*-axis. Based on such a configuration, the meta-atom can rotate the *x*-polarized incident waves to the *y*-polarized transmitted waves with high efficiency. Meanwhile, it can block the transmission of *x*-polarized waves incident from the back^1, 2^. As a result, asymmetric transmission can be achieved.

The phase responses can be controlled by adjusting the sheet admittance of the middle layer, which is determined by the geometric parameters of SSRR. Herein, we designed eight meta-atoms with different SSRRs in the middle layer using the genetic algorithms. The transmission phases of the eight meta-atoms cover the range of [0, 2π] with a spacing of π/4. To fulfill this purpose, we concerned three major geometric parameters of the SSRR: the open angle (*θ*) of the gap, the outer radius (*r*), and the width (*w*) of the split ring. Those parameters are schematically shown in Fig. S1a.

The design flow for optimizing these parameters is demonstrated in Fig. S1b. First, we carried out a rough parameter sweep using the full-wave simulation software. Then, we obtained a set of initial parameters. The meta-atom has a corresponding cross-transmission of $S_{21}^{M_{1}}$. In the simulation, the film thickness of gold structures is 200 nm, and the electric conductivity of gold is 4.56×10^7^ S m^-1^. The spacer sandwiched between the adjacent metallic layers is polyimide film with a thickness of 10 μm and a relative permittivity of 3.5×(1 - j0.0027). The substrate is 150 μm-thick sapphire with a relative permittivity of 9.4×(1 - j0.0004). The unit cell boundary condition is set in the simulation. On this basis, the other three SSRRs in the corresponding meta-atoms, which satisfy the requirements of equal cross-transmission amplitude and the π/4 phase difference between the adjacent meta-atoms, are designed by genetic algorithm one by one. While the remaining four SSRRs are obtained by rotating the first four SSRRs by 90° degrees in the *x-y* plane^3^. Considering the error in sample fabrication, we bounded the parameters using the following inequalities: 10 μm ≤ *r* ≤ 46 μm, 5 μm≤ *w* ≤40 μm, 5° ≤ *θ*≤ 90°, *w* ≤ (*r* – 3) μm.


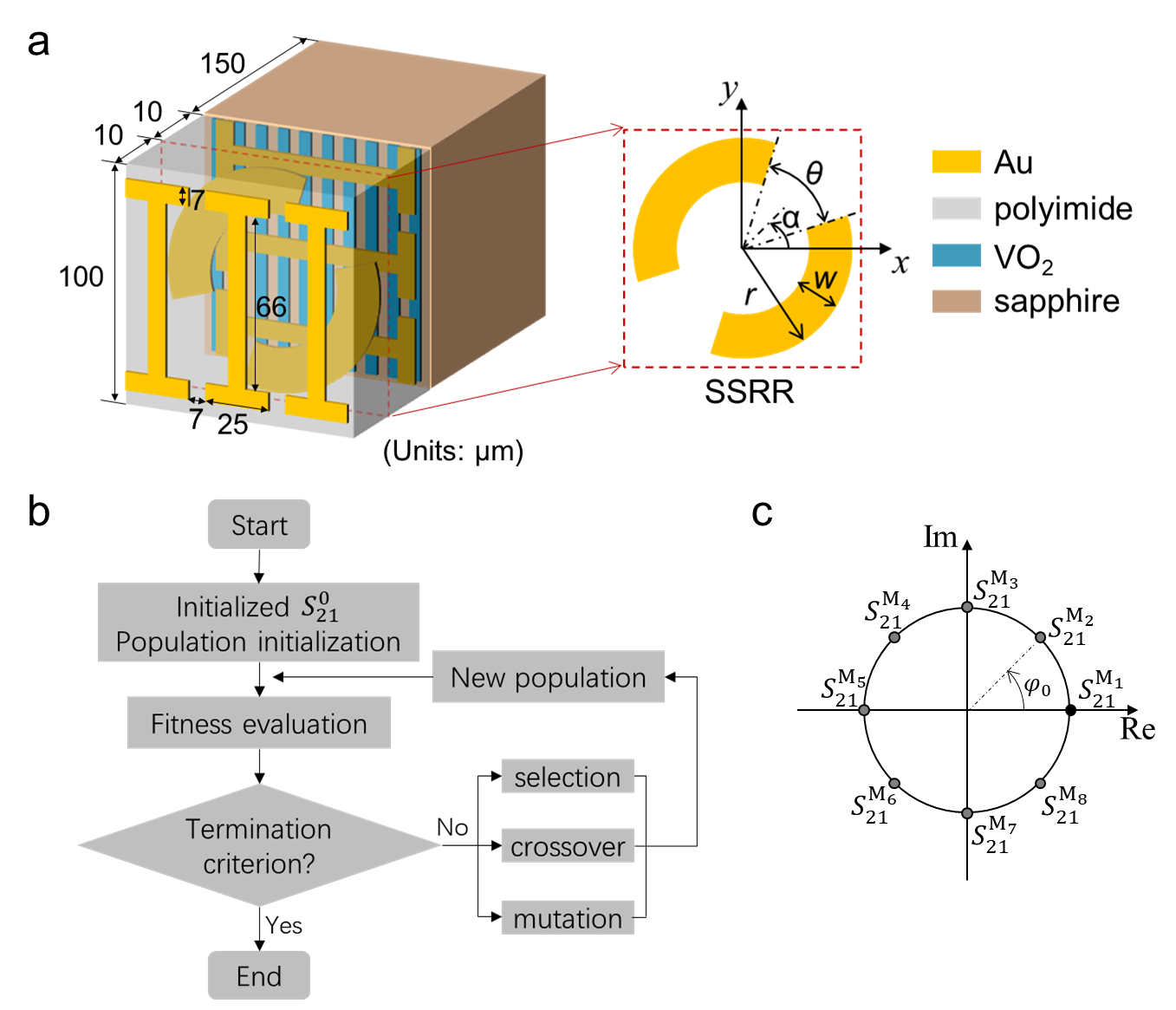


**Figure S1.** **Design and optimization process of eight meta-atoms.** (a) Diagram of VO_2_-integrated meta-atom and the corresponding SSRR in the middle layer. The geometric parameters are denoted in the figure. (b) Design flow of meta-atoms with the genetic algorithms. (c) Target cross-transmissivities of the eight meta-atoms at 0.99 THz denoted in the complex plane

To ensure the phase difference between the adjacent meta-atom is π/4, the fitness function adopted for genetic algorithms is

$fitenss=-\left| e^{j\left( \pi-\varphi_{0} \right)}S_{21}^{M}-S_{21}^{M_{1}} \right|/\left| e^{j\left( \pi-\varphi_{0} \right)}S_{21}^{M}+S_{21}^{M_{1}} \right|$ (S1)

where $S_{21}^{M}$ is the cross-transmissivity of the new individual at the target frequency, *φ*_0_ is the target phase difference between the desired meta-atom and the initial one whose phase response is *arg*($S_{21}^{M_{1}}$). Next, based on the inequalities mentioned above, the population with a size of 30 is initialized. Then, the fitness of the initial population is evaluated with Eq. S1. If the termination criterion is satisfied, the optimization procedure ends. Otherwise, the individuals with good fitness values are selected and then updated by the crossover and mutation to form the following populations. The design flow is shown in Fig. S1b. The optimization procedure works until the desired set of parameters is obtained. After the optimization, the desired meta-atom with the target phase difference (*φ*_0_) relative to the initial meta-atom has been found. The target cross-transmissivities of the eight meta-atoms with a phase interval of π/4 are denoted in the complex plane as schematically illustrated in Fig. S1c. The optimized geometric parameters of the SSRR in each meta-atom are listed in Table S1.

**Table S1. Geometric parameters of SSRR in different meta-atoms.**

| Meta-atom | *R* (μm) | *w* (μm) | *θ* (Deg.) | *α* (Deg.) |
| --- | --- | --- | --- | --- |
| M_1_ | 42 | 22 | 69 | 45 |
| M_2_ | 45 | 18 | 55 | 45 |
| M_3_ | 44 | 33 | 14 | 45 |
| M_4_ | 45 | 7 | 19 | 45 |
| M_5_ | 42 | 22 | 69 | -45 |
| M_6_ | 45 | 18 | 55 | -45 |
| M_7_ | 44 | 33 | 14 | -45 |
| M_8_ | 45 | 7 | 19 | -45 |

Section 2. Differences in the cross-transmission coefficient of the meta-atoms with and without insulating VO_2_.

The VO_2_ films used in our experiment have a thickness of around 200 nm and a non-negligible conductivity in the insulating state. Hence, the cross-transmission coefficient of the active meta-atom is slightly lower compared with that of the passive meta-atom without VO_2_ film. For verification, we simulated the cross-transmissions of the meta-atom of M_1_ in the cases without and with VO_2_ films. Figure S2 shows the simulated cross-transmission coefficient of the meta-atom M_1_ under the forward incidence of the *x*-polarized THz wave. The meta-atom with a conductivity of 1000 S m^-1^ has a lower cross-transmission amplitude relative to the passive meta-atom without the VO_2_ films. It can be attributed to the absorption of the VO_2_ film. However, the difference in the cross-transmission phases among these cases is negligible, which facilitates the realization of the meta-hologram by combining the passive and active meta-atoms together.


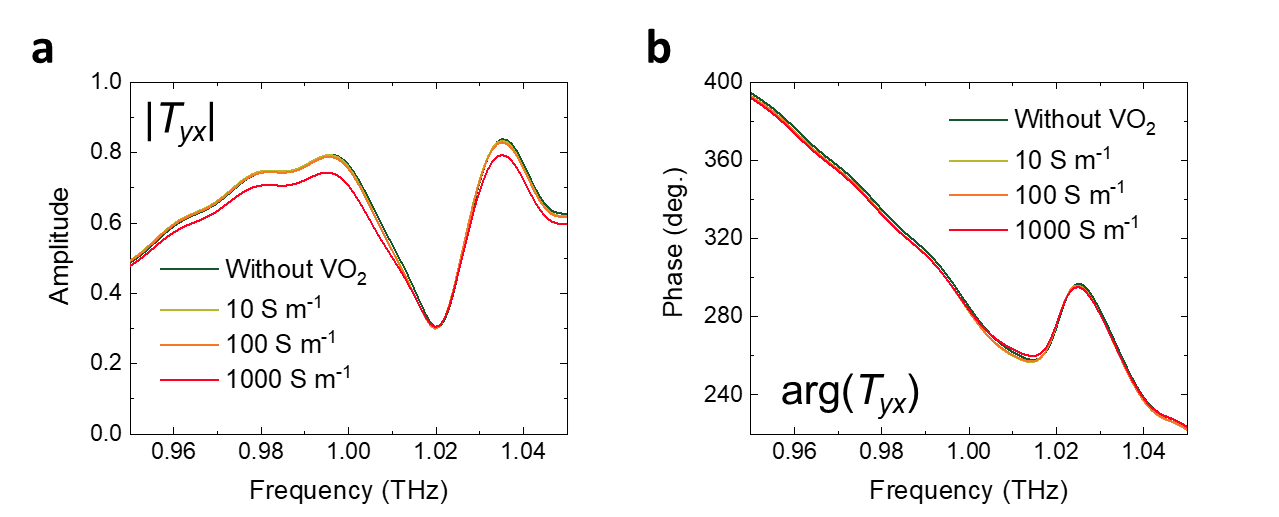


**Figure S2. Cross-transmission spectra of the meta-atom of M_1_.** Simulated cross-transmission amplitude (a) and phase (b) spectra of the meta-atom M_1_ in various configurations. The configurations for the simulation include the meta-atom without integration with VO_2_ film and three meta-atoms that are integrated with VO_2_ films with different conductivities

Section 3. Design principle of the meta-lens and meta-hologram.

Here, we supplement the design strategy that endows the reconfigurable Janus metasurface with the tunable focal length in the case of backward incidence. When the *x*-polarized THz wave is incident from the back, only the rotated meta-atoms modulate the phase of the cross-polarized transmitted wave with an amplitude of over 0.65, as shown in Fig. 2b of the main article. In Fig. 3b of the main article, those meta-atoms are identified by coloring the corresponding bottom-layered gold strips in blue.

The 60 × 60 meta-atoms were divided into 300 groups, each with 2 × 6 meta-atoms, and the meta-atoms in the group were labeled with ‘P_1_’, ‘P_2_’, ‘A_1_’, ‘A_2_’, ‘A_3_’, and ‘A_4_’, respectively. The meta-atoms of P_1_ and P_2_ do not integrate with VO_2_ films, and they are named passive elements. Meanwhile, the other meta-atoms are integrated with VO_2_ wire grids, and they are active elements. When VO_2_ enters the metallic state, the active meta-atoms are switched off with a significantly reduced cross-polarized transmission coefficient. In that case, only P_1_ and P_2_ contribute to the modulation of the wavefront of the *x*-polarized backward wave with the desired phase

$\varphi_{P_{1}\mathrm{or}P_{2}}=\frac{2\pi}{\lambda}\left[ \sqrt{x^{2}+y^{2}+f_{2}^{2}}-f_{2} \right]$ (S2)

where *f*_2_ is the focal length after the IMT of VO_2_, (*x*, *y*) are the positions of P_1_ and P_2_ at *z* = 0. When VO_2_ recovers back to the insulating state, A_2_ and A_4_ generate a complex cross-polarized transmission coefficient at *z* = *f*_2_ with the same amplitude but a phase shift of π relative to P_1_ and P_2_. Their destructive interference results in the erasure of the focal point at *z* = *f*_2_. Meanwhile, a new focal point is formed at *z* = *f*_1_ by phase profiles determined by A_1_ and A_3_. The phase shifts created by A_2_, A_2_, A_3_, and A_4_ are described by

$\varphi_{A_{2}\mathrm{or}A_{4}}=\frac{2\pi}{\lambda}\left[ \sqrt{x^{2}+y^{2}+f_{2}^{2}}-f_{2} \right]+\pi$ (S3)

$\varphi_{A_{1}\mathrm{or}A_{3}}=\frac{2\pi}{\lambda}\left[ \sqrt{x^{2}+y^{2}+f_{1}^{2}}-f_{1} \right]$ (S4)

In a word, the metasurface can tune the focal length for the backward waves based on the above principle.

Figure S3 illustrates the design principle of the reconfigurable Janus metasurface for thermally switched holographic images. For the forward illumination of the *x*-polarized wave, only half of the meta-atoms in which the metallic grating in the top layer is perpendicular to the polarization direction modulate the cross-polarized transmitted wave with the desired phase delays. The others block the THz wave, and those meta-atoms are marked with black, as shown in Figs. S3a, c. In addition, the active meta-atoms become opaque to incident THz wave after the IMT of VO_2_, and then those meta-atoms are also marked with black, as schematically shown in Fig. S3b. Hence, only the passive meta-atoms work to generate the desired holographic image at a predefined position after heating the metasurface.

The required phase profiles are calculated by the GS algorithm. The amplitude and phase distribution of the target holographic image are shown in Figs. S3e, h. The amplitude distribution forms the letter “U,” and the initial phase is all 0. When VO_2_ goes back to the insulating state, the active atoms in the metasurface generate a merged holographic image to switch the letter from “U” to “E,” as shown in Fig. S3f and S3i. When the sample is cool down to 25  C, both the passive and active atoms are working. The holographic image generated by the passive and active meta-atoms interferes, forming a new holographic image of the letter “E”.

Based on the above strategy, the letter “E” is independent of “U” and can be replaced by other letters or images. Numerical calculations using Rayleigh-Sommerfeld diffraction integral formula show that the proposed metasurface can switch the holographic image dynamically by changing the VO_2_ conductivity. Figures S3d and S3g show the theoretically-calculated holographic images when VO_2_ is in the metallic and insulating states, respectively.


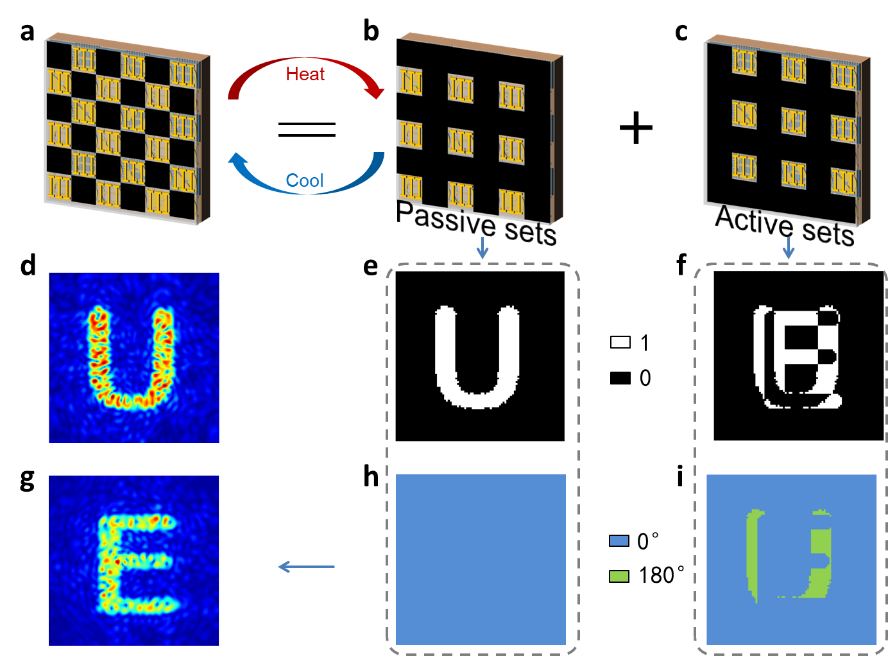


**Figure S3.** **Design strategy for the reconfigurable holographic images.** (a) Schematic of metasurface designed for the forward incident waves. It comprises passive (b) and active elements (c). When the metasurface temperature is above the phase transition temperature of VO_2_, only the passive elements modulate the *y*-polarized transmitted waves with the desired phase. They generated the holographic target image with an amplitude distribution of the letter “U” (e) and an initial phase distribution (h). For the generation of a new letter “E,” the active elements are required to create the holographic images with the amplitude of the merged letter (f) and a particular phase distribution (i). The phase of the part of the “U” pattern that overlaps the “E” pattern has a shift of π relative to the initial phase. Therefore, the overlapping part can be erased by destructive interference. With the design strategy, the metasurface can switch the holographic image from the letter “U” to “E” before and after the IMT of VO_2_. The corresponding numerical results are shown in (d) and (g).

Section 4. Fabrication process of reconfigurable Janus metasurface.

As illustrated in Fig. S4, the fabrication process of the proposed reconfigurable Janus metasurface is listed as follows.

1. Depositing 200 nm-thick VO_2_ film on the sapphire substrate by pulsed laser deposition.
2. Spin-coating the photoresist (AZ1500) film and patterning it using conventional ultraviolet photolithography.
3. Etching VO_2_ film to form wire grid structure by reactive ion etching.
4. Spin-coating the photoresist (AZ1500 and LOR10B) and forming the pattern using conventional ultraviolet photolithography.
5. Depositing titanium and gold films by radio frequency sputtering and forming the metallic structures in the bottom layer using the lift-off process.
6. Spin-coating the polyimide solution and baking it to form the polyimide layer.
7. Spin-coating the photoresist (AZ1500 and LOR10B) film and forming the pattern using conventional ultraviolet photolithography.
8. Depositing titanium and gold films by radio frequency sputtering and forming the metallic structures in the middle layer using the lift-off process.
9. Spin-coating the polyimide solution and baking it to form the polyimide layer.
10. Spin-coating the photoresist and forming the pattern using ultraviolet photolithography.
11. Depositing titanium and gold films by radio frequency sputtering and forming the metallic structures in the top layer using the lift-off process.


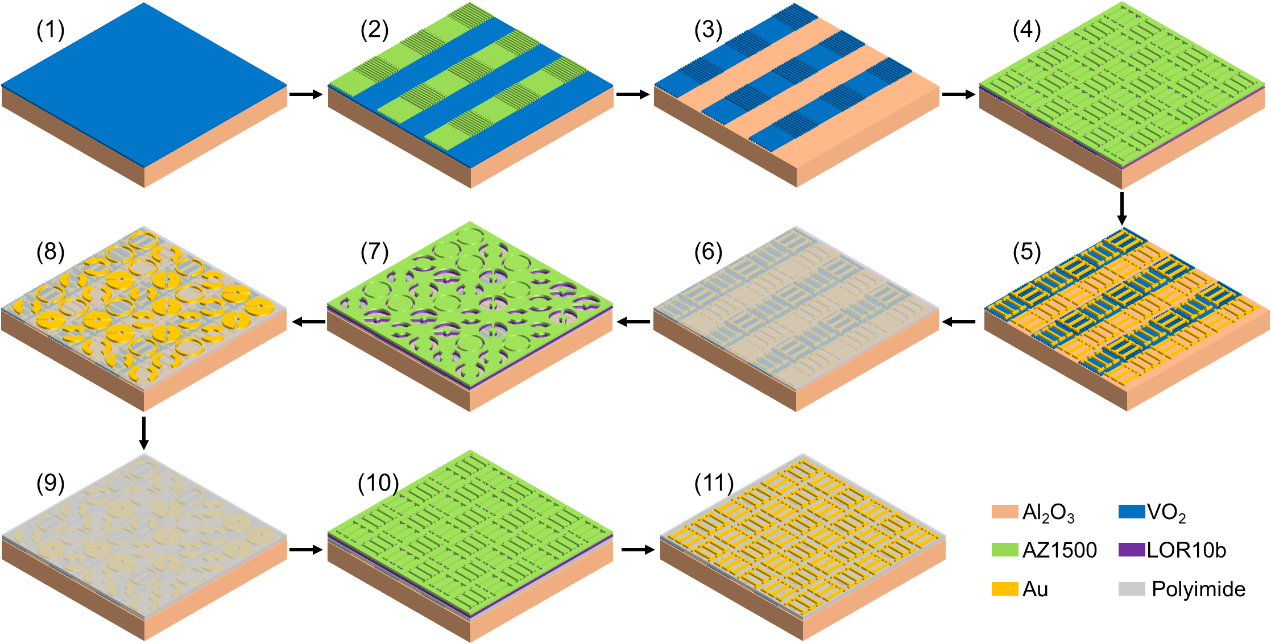


**Figure S4. Flow chart of the reconfigurable Janus metasurface fabrication process.**

Section 5. Discussion on the intensity of the focus generated by the meta-hologram at 85 ℃.

The *x*-directional cut of the measured focal point profiles in Fig. 4c of the main article shows that the focus intensity at 85 ℃ is lower than at 25 ℃, which is inconsistent with the fact that the two focal points are formed by the metasurface with the same effective aperture. As depicted in the subsection of *Dynamic Janus meta-lens*, at 85 ℃, the *y*-polarized transmitted wave is focused to the point *L*_2_ due to the phase modulation of the passive pixels P_1_ and P_2_ in each group, while the active pixels such as A_2_ and A_4_ do not modulate phases due to their zero cross-transmission amplitudes. However, in practice, the cross-transmission amplitude of those active pixels may not strictly be zero at 85 ℃. In that case, a portion of the THz wave would leak out from these pixels and coherently cancels out diffracted fields generated by P_1_ and P_2_ at the targeted focus due to their phase shift of π relative to P_1_ and P_2_.

For numerical analysis, we denote the cross-transmission amplitudes of the passive pixels (P_1_ and P_2_) and active pixels (A_2_ and A_4_) with $T_{yx}^{p}$ and $T_{yx}^{a}$, respectively. Based on the Rayleigh-Sommerfeld diffraction integral formula, we calculated the diffracted electric field *E_y_* along the *x*-direction under various combinations of $T_{yx}^{p}$ and $T_{yx}^{a}$ in Fig. S5. It can be seen that focal intensity at *L*_2_ (+1.50 mm, 0, 3.00 mm) at 85 ℃ decreases with $T_{yx}^{a}$, indicating that focus intensity is deteriorated by the THz wave leaked from the active pixels.


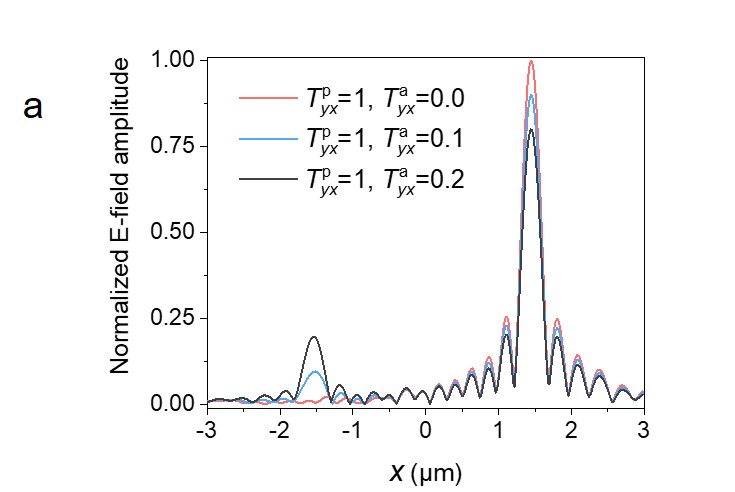


**Figure S5. Calculated diffracted electric field *E_y_* along *x*-direction under various combinations of** $\boldsymbol{T}_{\boldsymbol{yx}}^{\mathbf{p}}$ **and** $\boldsymbol{T}_{\boldsymbol{yx}}^{\mathbf{a}}$**.**

Section 6. Discussion on the difference among the reconstructed holographic images by experiments, simulations, and calculations.

Figure S6 compares the holographic images of “E” obtained from experiments, full-wave simulations, and theoretical calculations. The images obtained from full-wave simulations and experiments are much closer than the numerical results. One main reason is that the transmission amplitudes of the eight meta-atoms are identical in the theoretical calculation, and the phase interval is strictly π/4. In contrast, in the simulations or experiments, the coupling between the adjacent meta-atoms alters the transmission coefficient of each meta-atom, causing unequal transmission amplitudes and the deviation of phase interval from the designed value for eight different meta-atoms.

For further verification, we introduced random amplitude and phase errors in the numerical calculation. Hence, the transmission amplitude ($A_{i}$) and phase ($\varphi_{i}$) of the *i*-th meta-atom are determined by the following equations,

$A_{i}=0.8+\delta A\times\left( -1 \right)^{randi(2)}$ (S5)

$\varphi_{i}=\varphi_{i}^{\mathrm{ori}}+\delta P\times\left( -1 \right)^{\mathrm{randi}\left( 2 \right)}$ (S6)

where $\varphi_{i}^{\mathrm{ori}}$ is the initial phase of the *i*-th meta-atom without error. The function randi(2) is used to generate a random integer value range from 1 to 2, $\delta A$ and $\delta P$ denote the amplitude deviation and phase deviation, respectively.

For comparison, we illustrate the images of “U” obtained from the experiments, simulations, and theoretical calculations as shown in Fig. S6. Figures S6a and S6b are the experimental and simulated images, respectively. Both of them are copied from Fig. 5 in the main text. Figures S6c-6h are the theoretical results for various combinations of amplitude ($A_{i}$) and phase ($\varphi_{i}$). Figure S6 clearly shows that the holographic image of the letter “E” becomes blurred with the increase in the phase error *δP*. The electric field inside the outline of the letter “E” becomes weaker while the electric field outside the outline is enhanced. Such phenomenon is also observed in experimental and simulated results (Fig. S6a,b). From this point, the stray electric field that blurs the image can be attributed to the deconstructive interference induced by the phase error.


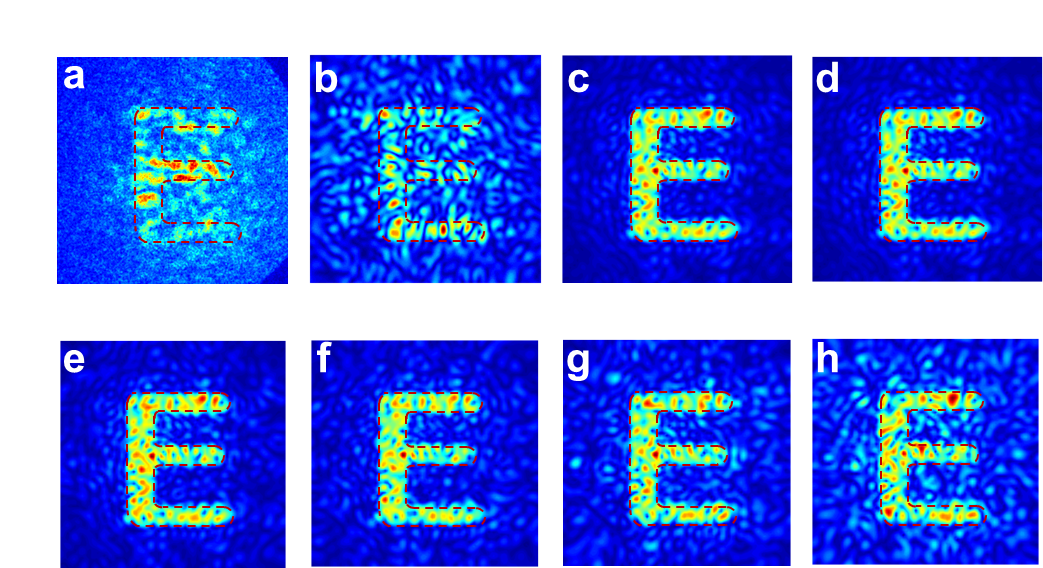


**Figure S6.** Holographic images of the letter “E” obtained from (a) experiments, and (b) simulations, theoretical calculations with the parameter combination of (c) *δA* = 0, *δP*=0°; (d) *δA* = 0.2, *δP* = 0°; (e) *δA* = 0.2, *δP* = 10°; (f) *δA* = 0.2, *δP* = 20°; (g) *δA* = 0.2, *δP* = 30°; (h) *δA* = 0.2, *δP* = 40°.

Section 7. Holographic images generated by the fabricated Janus meta-hologram when illuminated with the *y*-polarized polarized wave.

As discussed in the main text, the meta-atoms consisting of the meta-hologram have asymmetric but reciprocal transmission. Hence, according to the reciprocity theorem, the distribution of the transmitted *x*-polarized field generated by the meta-hologram in the case of *y*-polarized backward incidence is the same as the *y*-polarized transmitted field when the *x*-polarized forward wave is incident. To verify this, we carried out the full wave simulation on the meta-hologram to achieve the *x*-polarized transmitted field in the case of *y*-polarized incidence, as shown in Fig. S7. Besides, the simulated *y*-polarized transmitted field achieved in *x*-polarized incidence is copied from Fig. 5 of the main article. The holographic image (*x*-polarized electric field) of the letter “N” obtained in the case of *y*-polarized forward incidence can be reconstructed by illuminating the meta-hologram with the *x*-polarized plane wave from the backward direction, which is consistent with the reciprocity theorem.


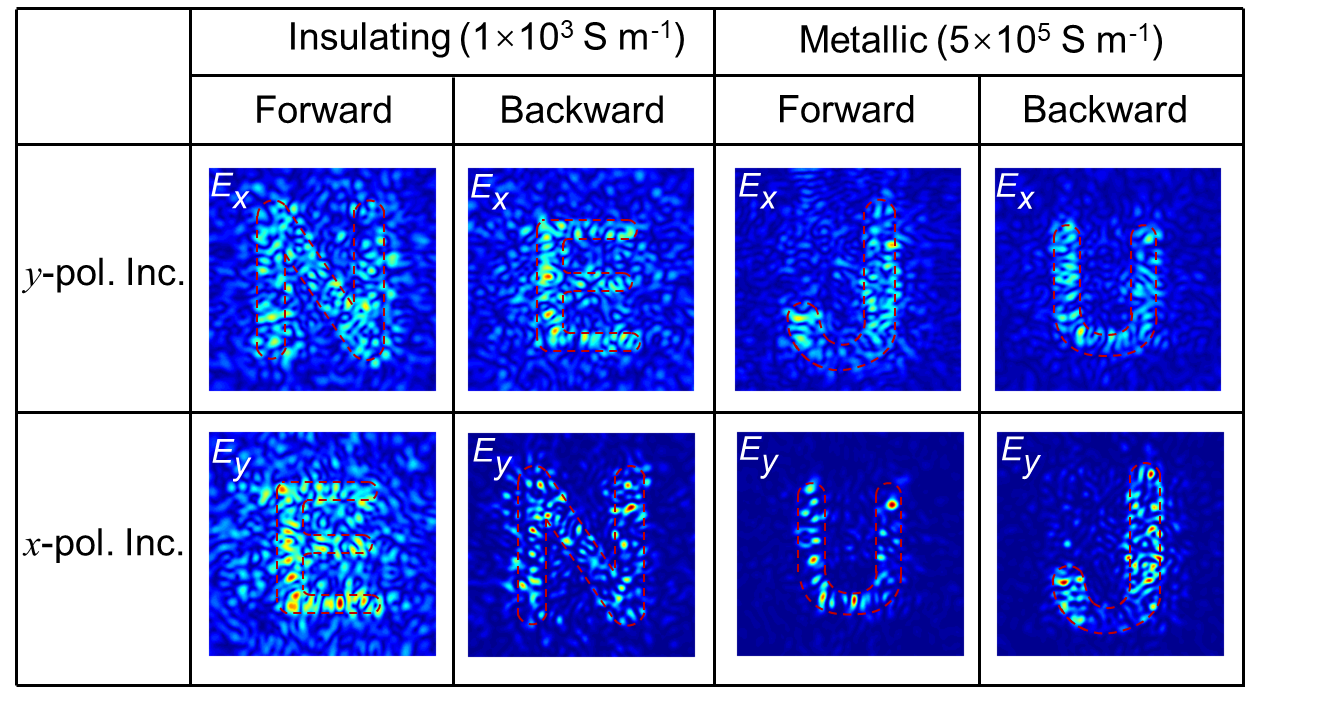


**Figure S7. Simulated holographic images when the meta-hologram is incident by the *x*-/*y*-polarized THz wave (0.99 THz) from the backward/forward direction.**

Section 8. Demonstration of the proposed metasurface as a beamformer in THz communication.

In recent years, THz communication has become one of the key development direction of next-generation communication, and the beamforming technology is critical to improving the coverage of THz communication. On the one hand, the mature beamforming technologies widely used in the microwave band are no longer effective at THz frequencies. On the other hand, the emerging reconfigurable metasurface with a flexible wavefront modulation capability has become a good choice for THz beamforming. In THz communication applications, many low-cost metasurface-based beamformer must be arranged at different locations to shape the communication beams. The compact size and the simple fabrication process of the metasurfaces proposed in this work make them possible for large-scale applications. The direction-multiplexing can effectively reduce the number of metasurface scatters, and thus, the cost may be effectively reduced. What is more, the reconfigurability makes the beam steering more flexible. Therefore, we think the demonstration of the proposed metasurface as the beamforming antenna will be a high-impact application in the future. Figure S8 schematically shows the potential applications of the proposed Janus metasurface in THz wireless communication.


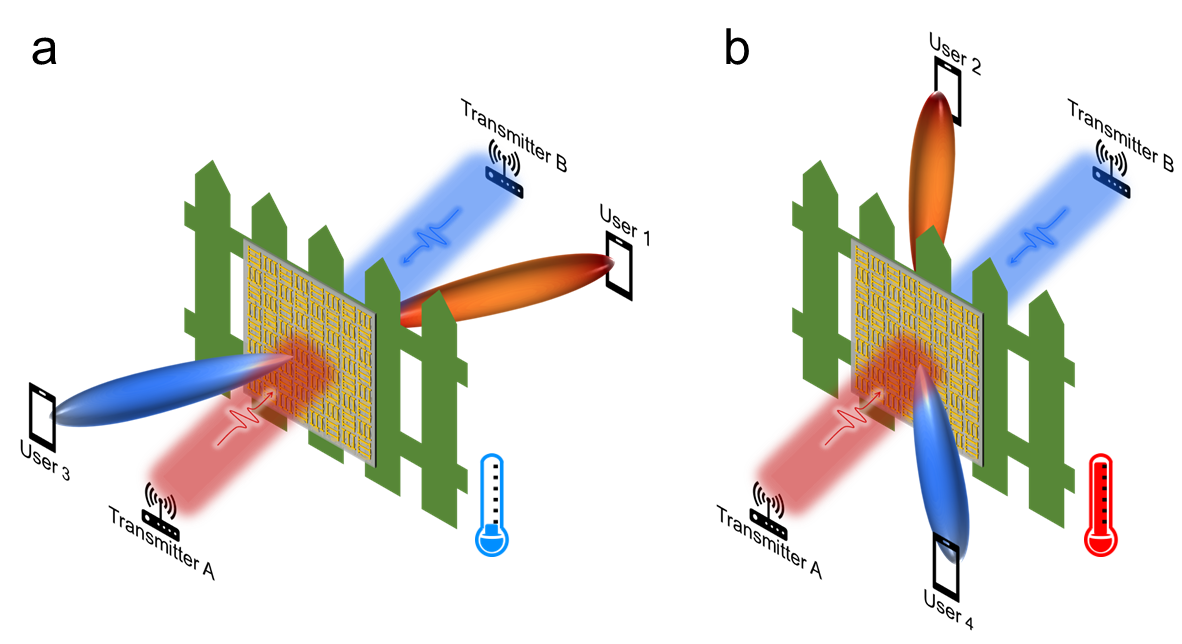


**Figure S8. Schematic of applying the proposed metasurface in THz communication.** (a) Before the IMT of VO_2_ films, the metasurface can deflect the forward-incident THz wave of Transmitter A to User 1 while deflecting the backward-incident THz wave from Transmitter B to User 3 due to the direction-dependent property. (b) After the IMT of VO_2_ films, the THz beam for wireless communication can be deflected to others users.

To realize the tunable direction-dependent THz beam steering, we numerically analyzed the electric far-field distribution of the metasurface with various phase profiles in Fig. S9. Here, the meta-atoms for the forward and backward incidences are alternately arranged along the *y*-axis direction as shown in Fig. S9a. In each group of 2 × 3 meta-atoms, P_1_ and A_1_/A_2_ are the passive and active meta-atoms, respectively. After the insulator-metal transition (IMT) of VO_2_, the active meta-atoms block the cross-transmission completely. In that case, the beam deflection direction of the transmitted THz wave is determined only by P_1_. According to the generalized Snell's law, the phase profiles of P_1_ in each row should satisfy the following equation to make the forward incident wave deflect to the angle of -10°,

$\varphi_{P}\left( x \right)=\frac{2\pi}{\lambda}x\sin{(-10}^{\circ})$ (S7)

where the *λ* is the wavelength of the incident THz wave in the free space, and *x* is the position of P_1_ along the *x*-direction.

When the VO_2_ state recovers from the metallic state to the insulating state, A_1_ and A_2_ modulate the forward incident wave with the predefined phase. To suppress the beam deflection at -10° which is determined by P_1_, the phase profiles of A_1_ satisfy the following relationship

$\varphi_{A_{1}}\left( x \right)=\varphi_{P}\left( x-D \right)+\frac{2\pi}{\lambda}D\sin{(-10}^{\circ})+\pi$ (S8)

where *D* is the distance between the P_1_ and A_1_. The meta-atom size in the demonstration is 50 μm 🞨 50 μm. The beam at -10° will be suppressed due to the destructive interference. A_2_ alone determines the newly generated beam. The modulated phase caused by these meta-atoms is

$\varphi_{A_{2}}\left( x \right)=\frac{2\pi}{\lambda}x\sin{(30}^{\circ})$ (S9)

Following the above strategy, the phase profiles for the backward incident wave can be arranged to deflect the incident wave to the angle of 50° before the IMT and -40° after the IMT, as demonstrated in Fig. S9b. Thus, using the proposed metasurface, THz beam steering with predefined deflection angles for two different incident directions is realized.


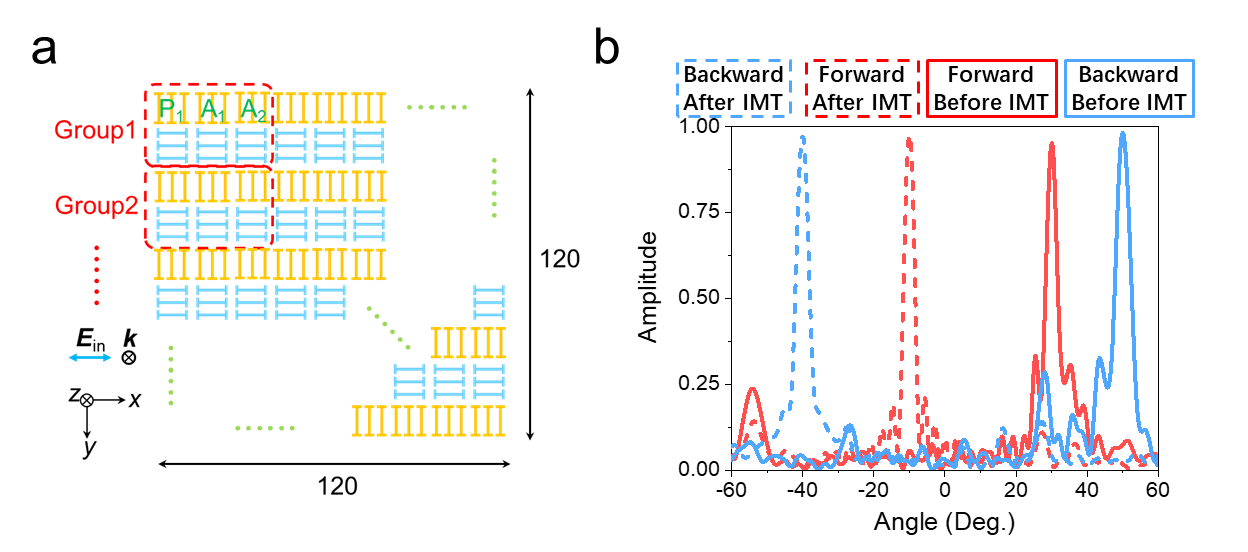


**Figure S9. Schematic of the top-layered metallic pattern for THz beam steering.** (a) Schematic of the top-layered metallic pattern. The meta-atoms designed for the forward incidence are colored yellow, while others for backward incidence are colored blue. The metasurface consists of 120 × 120 meta-atoms. The periodicity of the meta-atom is 50 μm. (b) Numerically calculated far-field distribution of the metasurface with different VO_2_ states and incident directions.

Section 9. THz spectral imaging system for the characterization of the Janus metasurface.

Figure S10 shows the schematic diagram of the THz spectral imaging system. The laser pulse generated from the 800-nm femtosecond laser is divided into the pump and probe light by a beam splitter. The wavefront of the pump light is tilted using a grating and two cylindrical lenses (L1 and L2). Then, the pump light is incident on the LiNbO_3_ crystal. The THz pulse is generated using the optical rectification effect in the nonlinear crystal. To ensure that the incident THz wave is *x*-polarized and the transmitted wave detected by the GaP detection crystal is *y*-polarized, we used two THz polarizers in front and back of the sample, respectively, as shown in Fig. S10. Based on the Pockels effect, the transmitted THz electric field strength is detected by the birefringence of the probe light in the GaP crystal. The probe light reflected by the GaP crystal becomes elliptically polarized after passing through the quarter-wave plate and is further divided into two orthogonally polarized components by the polarized beam splitters. Finally, the probe light carrying THz electric field distribution in two orthogonally polarized components is captured by a near-infrared charge-coupled device (CCD). By scanning the optical delay line, the time-domain signals of each pixel in the CCD camera are sampled. After the Fourier transforms, the spatial distribution of the THz field at different frequencies is obtained.


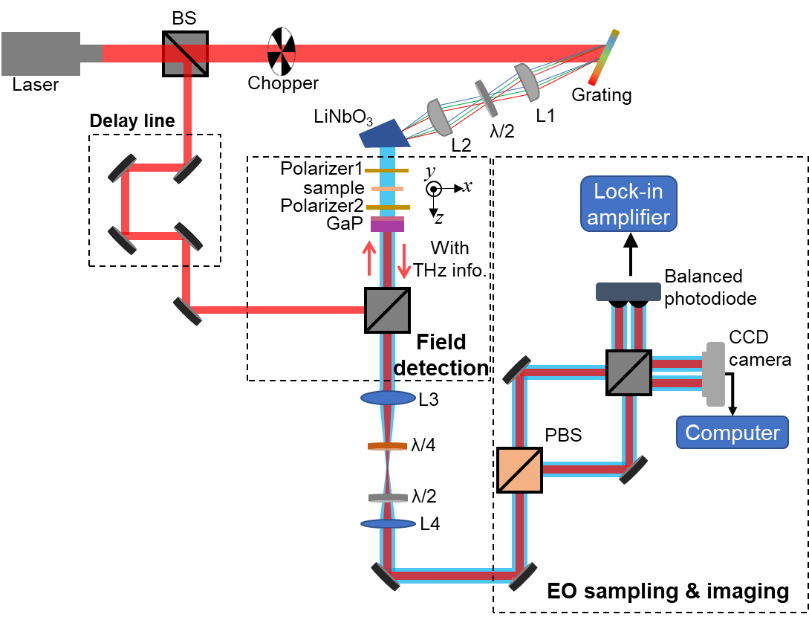


**Figure S10. Schematic diagram of the THz spectral imaging system.**


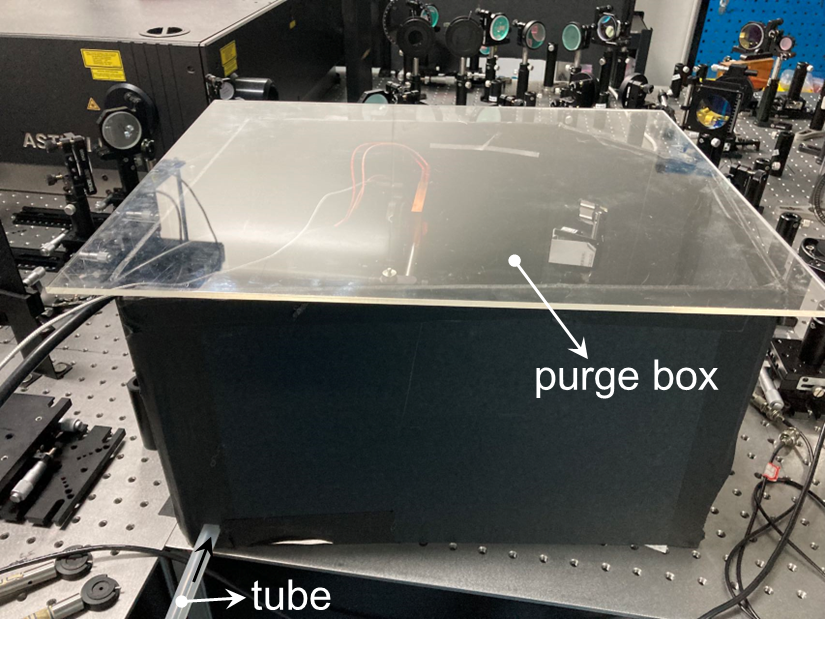


**Figure S11.** **Photo of self-made dry-air purge box that encloses the THz beam path.**


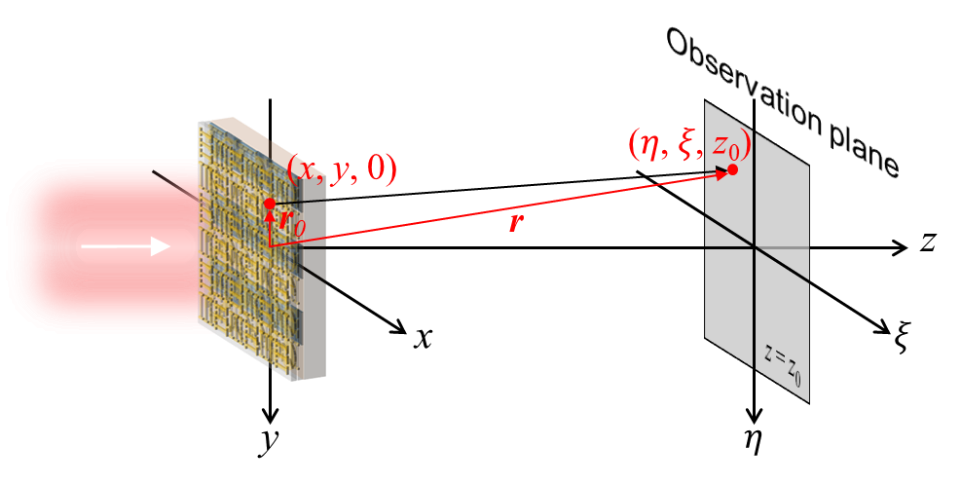


**Figure S12. Illustration of the coordinate system for Rayleigh-Sommerfeld diffraction calculation.**

Supplementary References

[S1] Pfeiffer, C. et al. High performance bianisotropic metasurfaces: asymmetric transmission of light. *Physical Review Letters* **113**, 023902 (2014).

[S2] Chen, K. et al. Directional Janus metasurface. *Advanced Materials* **32**, 1906352 (2020).

[S3] Wu, L.-X. et al. Transmissive metasurface with independent amplitude/phase control and its application to low-side-lobe metalens antenna. *IEEE Transactions Antennas Propagation* **70**, 6526-6536 (2022).
